# High Interannual Variability in Connectivity and Genetic Pool of a Temperate Clingfish Matches Oceanographic Transport Predictions

**DOI:** 10.1371/journal.pone.0165881

**Published:** 2016-12-02

**Authors:** Maria Klein, Sara Teixeira, Jorge Assis, Ester A. Serrão, Emanuel J. Gonçalves, Rita Borges

**Affiliations:** 1 CCMAR - Centre of Marine Sciences, University of Algarve, Campus de Gambelas, 8005–139 Faro, Portugal; 2 MARE - Marine and Environmental Sciences Centre, ISPA – Instituto Universitário, R. Jardim do Tabaco 34, 1149–041 Lisbon, Portugal; Department of Agriculture and Water Resources, AUSTRALIA

## Abstract

Adults of most marine benthic and demersal fish are site-attached, with the dispersal of their larval stages ensuring connectivity among populations. In this study we aimed to infer spatial and temporal variation in population connectivity and dispersal of a marine fish species, using genetic tools and comparing these with oceanographic transport. We focused on an intertidal rocky reef fish species, the shore clingfish *Lepadogaster lepadogaster*, along the southwest Iberian Peninsula, in 2011 and 2012. We predicted high levels of self-recruitment and distinct populations, due to short pelagic larval duration and because all its developmental stages have previously been found near adult habitats. Genetic analyses based on microsatellites countered our prediction and a biophysical dispersal model showed that oceanographic transport was a good explanation for the patterns observed. Adult sub-populations separated by up to 300 km of coastline displayed no genetic differentiation, revealing a single connected population with larvae potentially dispersing long distances over hundreds of km. Despite this, parentage analysis performed on recruits from one focal site within the Marine Park of Arrábida (Portugal), revealed self-recruitment levels of 2.5% and 7.7% in 2011 and 2012, respectively, suggesting that both long- and short-distance dispersal play an important role in the replenishment of these populations. Population differentiation and patterns of dispersal, which were highly variable between years, could be linked to the variability inherent in local oceanographic processes. Overall, our measures of connectivity based on genetic and oceanographic data highlight the relevance of long-distance dispersal in determining the degree of connectivity, even in species with short pelagic larval durations.

## Introduction

Adults of most marine benthic and demersal fish are site-attached, with the dispersal of their larval stages ensuring connectivity (e.g. [1]). Due to difficulties in tracking small larvae and because early connectivity studies focused on larvae of temperate species with poor swimming capabilities [2, 3], initial research considered fish larvae as passive particles vulnerable to large current systems [4]. Such studies revealed the existence of long-distance dispersal, resulting in highly connected open populations (e.g. [5, 6]). In contrast, a growing number of studies have recently shown significant levels of self-recruitment and reduced dispersal distances in many tropical (e.g. [7, 8, 9]) and a few temperate fish species (e.g. [10, 11]). These studies, combined with findings of persistent nearshore larval distributions of coastal species [12, 13] and increasing evidence of active larval behavior [14, 15, 16], show that larval dispersal can be restricted and populations can be more demographically closed over ecological time scales than previously assumed. Although ocean currents are a major factor affecting larval transport (e.g. [17, 18]), local conditions may favor reduced dispersal distances and larval retention [19, 20]. Some fish larvae significantly interact with currents by migrating vertically, changing their swimming speed and direction to actively find settlement habitats (e.g. [14, 16, 21]).

Several types of methods have been developed to overcome the knowledge deficit in marine fish larval connectivity. Indirect approaches include genetic connectivity studies (e.g. [22, 23]), microchemistry of otoliths as geochemical markers (e.g. [24, 25]) and biophysical models, which are used to reconstruct dispersal tracks, population connectivity and to identify potential sink and source populations (e.g. [26, 27]). Direct approaches include mark-recapture methods with stable barium isotopes or tetracycline immersion (e.g. [8, 28]), assignment tests [29] and parentage analysis with highly polymorphic genetic markers [30, 31].

Estimating the degree of dispersal and connectivity among populations is essential to understand persistence and resilience of populations, a critical question in conservation ecology, particularly relevant for marine protected areas [32, 33]. Focusing on a major marine protected area in Portugal, the Marine Park of Arrábida (MPA), a good model species to study these questions of connectivity within and out of the MPA, is *Lepadogaster lepadogaster*, a temperate clingfish, for which there is evidence from a combination of biological, ecological and oceanographic features, suggesting high population structure and low connectivity among populations. These features include short pelagic larval duration (PLD [34, 35, 36]), the occurrence of all larval developmental stages in the nearshore environment [12, 37], the hatching of well-developed larvae [35] and oceanographic data suggesting retention in the region of this MPA [38].

In this study we address the broad question of whether populations of marine species function more as self-recruiting units or as open interconnected networks, focusing on a model expected to represent the former case. We focus particularly on trying to understand whether the answer to this question is consistent over time and whether the temporal variability can be explained by oceanographic transport patterns. Specifically, we aim to test the predictions of high population structure and low connectivity among populations of *L*. *lepadogaster* at the spatial scale of a marine protected area and the adjacent coastlines. We apply genetic tools to answer this question and compare the results with simulations of particle transport by marine currents to evaluate the hypothesis that temporal variability in genetic connectivity might depend on oceanographic variability.

## Material and Methods

### Study Species

The shore clingfish *Lepadogaster lepadogaster*, Bonnaterre (1788) a Gobiesocidae, is a small cryptobenthic temperate reef fish, strictly restricted to smooth boulders and stones with little biological cover in the intertidal zone of rocky coasts [39, 40]. It occurs from the British Isles to northwestern Africa, including the Mediterranean and Black Sea [39, 41]. Egg clutches attached to rocks can contain 60 to 265 eggs [41]. Males aerate and rub the eggs until hatching [35]. *L*. *lepadogaster* has an embryonic development of 16 days and hatches well-developed larvae with an open mouth and anus, fully pigmented eyes and with lengths of 5.2–5.3 mm [35]. The PLD of *L*. *lepadogaster* lasts 11–18 days [34, 35]; comparably short in relation to other temperate rocky reef species [36].

### Study area and sampling

The MPA, in central Portugal, was the location selected for this study since several rocky reef fish species there occur very near-shore and near-reef, indicating potential for larval retention close to the adult habitat [12, 13, 37]. It can be regarded as a continental island since it is separated from the nearest rocky reef systems by 40 km (to the north) and 60 km (to the south) of sandy coastline. Additionally, this region is oceanographically retentive with reduced offshore advection [38].

**Fig 1:**
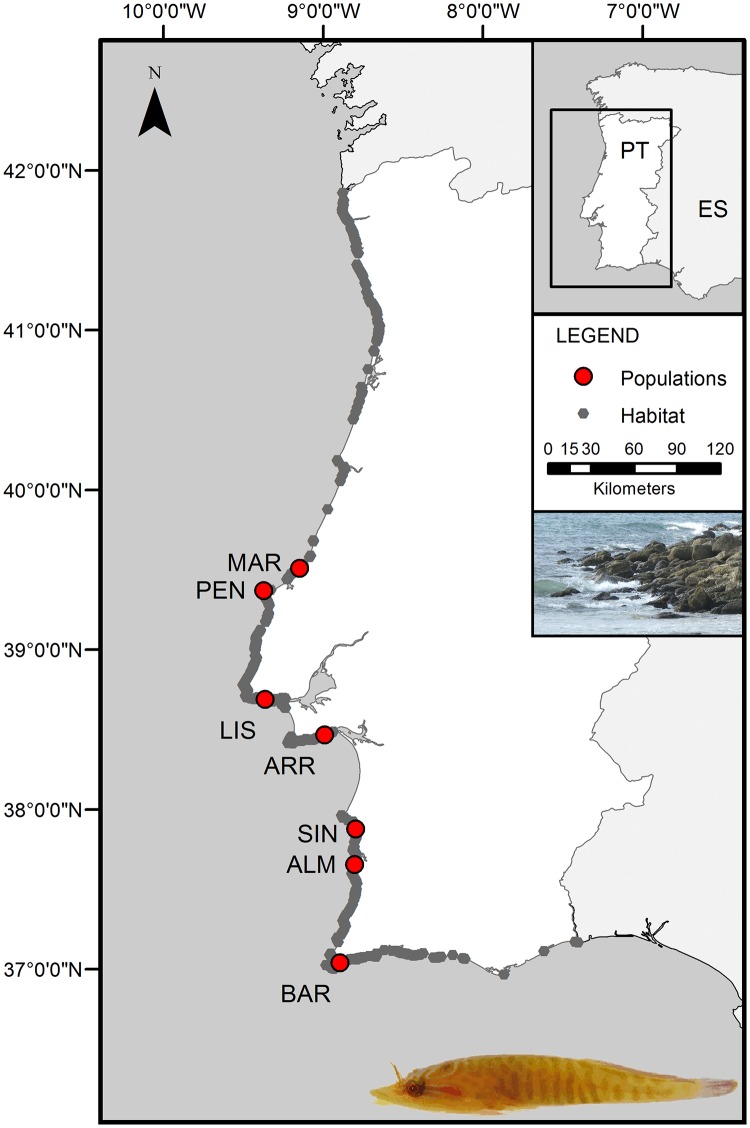
Study area. Sampling sites are shown as red points (with site labels as in Table 1) and possible habitat sites of *L*. *lepadogaster* in grey, selected by the presence of boulders in intertidal rocky reefs. Images on the right side show an adult fish (photo by P. Coelho) and a typical habitat site. Country maps were provided by the Portuguese Institute of Hydrography [42].

In 2012, adult fish were sampled at a focal site (ARR) within the MPA and in areas to the north and south. Northerly sites included rocky coasts close to Lisbon (LIS), Peniche (PEN) and São Martinho do Porto (MAR), while southerly sites included Sines (SIN), Almograve (ALM) and Barranco (BAR) (Fig 1, Table 1). Adults were also collected in ARR and LIS in 2011. Recruits were collected only in ARR, both in 2011 and 2012. In total, the sampling area encompassed 390 km of coastline and sites were separated by an average of 65 km.

**Table 1:** Summary statistics of sampled adult and recruit populations from 2012 and 2011.

Pop_ID	Site	Adults/ Recruits	Year	N	% P	Mean SL ± SD	*A*	*A*_*rich*_	*H*_*E*_	*H*_*O*_	*F*_*IS*_
Mar_A_12	MAR	adults	2012	39	20.6	54.9±6.4	9.17	8.23	0.75	0.71	0.05*
Pen_A_12	PEN	adults	2012	39	16.0	54.2±5.2	9.58	8.25	0.74	0.68	0.08***
Lis_A_12	LIS	adults	2012	40	54.9	47.4±5.8	8.42	7.47	0.72	0.63	0.13***
Arr_A_12	ARR	adults	2012	40	13.9	52.8±4.1	9.00	8.14	0.75	0.64	0.14***
Sin_A_12	SIN	adults	2012	40	9.3	45.3±6.0	9.92	8.58	0.75	0.7	0.06**
Alm_A_12	ALM	adults	2012	24	43.3	45.3±6.0	7.83	7.83	0.75	0.69	0.08**
Bar_A_12	BAR	adults	2012	36	7.0	37.0±5.6	9.75	8.85	0.75	0.68	0.08***
Lis_A_11	LIS	adults	2011	30	41.2	56.1±4.5	10.33	10.04	0.81	0.74	0.09***
Arr_A_11	ARR	adults	2011	29	10.1	51.3±7.0	8.92	8.55	0.76	0.66	0.13***
Arr_R_12	ARR	recruits	2012	40	-	18.6 ± 4.1	9.50	8.32	0.73	0.56	0.23***
Arr_R_11	ARR	recruits	2011	39	-	17.9 ± 4.1	9.17	8.27	0.73	0.62	0.16***

Legend: Population label (Pop_ID), Site abbreviation as in Fig 1, Number of individuals sampled (N), estimated proportion of population sampled (% P), mean standard length ± standard error (Mean SL ± SD), mean number of alleles across loci (A), mean allelic richness (A_rich_), expected (H_E_) and observed (H_O_) heterozygosities and inbreeding coefficient (*F*_*IS*_). Significance levels are indicated (*p<0.05, **p<0.01, ***p<0.001).

Adults and juveniles were collected at low tide during spring tides under boulders and stones in large boulder fields and in tide pools between rocky platforms. Adult fish were sampled before the recruitment season (February-June), while recruits were collected in autumn after recruitment (August-September). In order to roughly estimate population size, the density of *L*. *lepadogaster* at ARR was determined via a field survey of all suitable boulder habitat, and this was extrapolated to the other sites. ARR was chosen for this field survey because of its suitable habitat and high fish density.

### Ethics statement

For this study, approximately a 0.03 cm^3^ tissue sample from the dorsal fin was clipped from adult *L*. *lepadogaster* and stored in 96% ethanol. Adult fish were released directly back into the same location where they were collected. Several adult fish were placed into aquaria after this procedure; no mortality due to handling was observed and fin regeneration occurred within weeks. At ARR, juvenile fish were euthanized and fixed in 96% ethanol. *L*. *lepadogaster* is not a species of conservation concern (IUCN, 2014: Least Concern) and thus no specific permits were required, except for sampling at ARR. This permit was issued by the Portuguese Nature Conservation Institute (ICNF).

In 2011 and 2012, when the field work of this study was carried out, the ethics legislation that existed in Portugal (Diário da República, decree 1005/1992) did not specify methods of euthanasia for fish. An ethics committee approval for the animal procedure was not required and animal suffering was considered negligible when immersing juvenile fish in ethanol as death occurred quickly (1–2 sec). Since 2013, an approval for animal procedures by a specific ethic committee is required, and specific euthanasia procedures are regulated (decree 113/2013).

Genomic DNA from fin- (adults) and muscle tissue (juveniles) was extracted as in [39], excluding the phenol/chloroform purification step. Fifteen microsatellite loci were amplified and analyzed, as described in Teixeira et al. [43], but with higher primer specific annealing temperatures for most loci. Due to low polymorphism and some amplification inconsistency, only 12 of the initial 15 loci were used for data analysis.

### Data Analysis

#### Genetic diversity

The mean number of alleles across loci (A), expected (H_E_) and observed (H_O_) heterozygosity, and the inbreeding coefficient (*F*_*IS*_) were determined with GENETIX, 4.05 [44]. Standardized allelic richness was estimated in R 3.1 [45] using the R package standARich vl1.00 [46]. Correction of p-values for multiple comparisons was performed using the false discovery rate in QVALUE [50].

#### Population structure and connectivity

GENETIX was used to estimate *F*_*ST*_, pairwise population differentiation, for each locus and over all loci. The probability of the F statistic being greater than zero was calculated with a permutation approach (10,000 replicates). Additionally, significant differentiation was confirmed by 95% confidence intervals (CI) above zero [47], calculated using the R package “diversity”. The software FreeNA [48] was used to detect null alleles and to compare global and pairwise *F*_*ST*_ between our raw microsatellite data and a corrected version after applying the excluding null alleles (ENA) correction method with 1,000 bootstrap repetitions. Furthermore, Jost’s D, a differentiation index that is not affected by the number of alleles per locus [49, 50], were estimated (averaged over loci) with the R package “DEMEtics” [51]. Here, p-values were obtained by 1,000 bootstrap resampling. Correction of p-values for multiple comparisons were performed using the false discovery rate in QVALUE [52].

Isolation-by-distance (IBD) analyses were conducted for both pairwise *F*_*ST*_ and Jost’s D-values independently in R by using the mantel.randtest function in the R package ade4 and 10,000 permutations. For these analyses, shoreline distance was calculated with the R-package “gdistance” [53].

The software STRUCTURE version 2.1 [54] was used to assign individual genomes to a number of genetic populations estimated to minimize Hardy-Weinberg and Linkage disequilibrium. Analysis was conditioned with an initial burn-in of 50,000 cycles followed by 100,000 additional cycles using an admixture ancestry model with correlated allele frequencies. Runs for each K (1 to 8) were repeated 20 times. Both Pritchard’s [54] L(K) criterion and the delta K criterion of Evanno et al. [55] were applied using STRUCTURE HARVESTER [56]. The software CLUMPP (version 1.1.2) was used to align the 20 replicate cluster analyses for the chosen K, by using the Greedy algorithm [57]. Results of CLUMPP were then visualized with the software DISTRUCT (version 1.1; [58]).

A Discriminant Analysis of Principal Components (DAPC) was also used to infer population structure. A DAPC can assign individual genotypes to predefined groups using different criteria than STRUCTURE, while being as sensitive [59]. The dapc function within the R package adegenet performs a Principal Component Analysis (PCA) using the dudi.pca function from the ade4 package and a linear discriminant analysis (DA) with the lda function from the MASS package [59]. For our data set 53 PCs, comprising 87% of the genetic information, were retained (Fig A in S3 File).

#### Population and parentage assignment

To assign or exclude recruits to/from the 7 sampled populations, we used a partial Bayesian classification method [60] in GENECLASS 2 [61] together with a Monte Carlo re-sampling method [62]. All adult fish (N = 317) from the sampled populations were used as reference and 10,000 genotypes were simulated from these reference populations for the Monte Carlo algorithm. A recruit was assigned to a population if its probability of assignment was >0.05 for only one population. A recruit was determined as immigrant if the probabilities of assignment were below 0.05 for all populations [63].

To further infer the possible origin of the parents and offspring, we used the maximum likelihood approach implemented in COLONY v.2.0 [64, 65], which assigns parentage from individual multilocus genotypes. Recruits (sampled at ARR in 2011, 2012) were used as offspring, while all adult populations were used as candidate male and female parents. The rate of allele dropout used was 0.05 and the rate of other errors was 0.01 [65]. A full-likelihood method was used. We assumed polygamous mating systems for both males and females since in previous aquaria experiments it was observed that females spawn several times during the spawning season with different males and males have eggs from different females. The analysis was run four times and only relationships with a probability higher than 0.95 were considered. Both methods were used to estimate self-recruitment in ARR, the proportion of local recruits that come from local parents [66] and to infer dispersal trajectories along sampled populations.

#### Dispersal model

Estimates of population connectivity of *L*. *lepadogaster* were inferred by developing individual-based Lagrangian Numerical Simulations (LNS; as reviewed by [26, 67]) using early life history parameters that are known for this species. The experiments were performed following the framework implemented by Assis et al. [27], utilizing daily data derived from the Hybrid Coordinate Ocean Model (HYCOM), a high-resolution hind cast of three-dimensional velocity fields forced by wind stress, wind speed, precipitation and heat flux [68]. This model is able to resolve oceanic filaments, fronts, meandering currents and eddies, some key processes needed to accurately simulate dispersion of drifting larvae [68, 69].

Individual particles simulated drifting larvae of *L*. *lepadogaster* by incorporating a maximum PLD of 18 days [34] and a set of state variables such as location (longitude and latitude), age (day), developing stage (competent or non-competent for settlement) and status (dead or alive; dead are considered particles that have not found habitat for settlement after 18 days). Suitable habitats [39] along the SW Iberian Peninsula were quantified using a Geographic Information System (GIS) and gridded to match 0.05° (~9 km^2^) spatial resolution. Particles were released from each cell every 6 hours during the spawning season of *L*. *lepadogaster* (from March to August; [39] and pers. observation) and allowed to drift for the maximum PLD. The geographical position of each particle was determined every hour (24 steps per day) using bilinear interpolation of velocity fields, while integrating in the path equation a 4th Order Runge-Kutta adaptive time-step (e.g. [69]).

Contrasting transport experiments (passive vs. vertical migration) were performed based on the early life history of this species. In the passive transport experiments, particles used the surface (0 meters) or the bottom (10 meters) layers independently. In the vertical transport experiments, particles were allowed to move from the surface to the bottom layers on the 10^th^ day of PLD, which is when the larvae acquire competence for settlement [35, 70]. Additional parameters such as mortality rate and population sizes were not included, since this information is not available for *L*. *lepadogaster*. This model was used to estimate potential distances and patterns of dispersal and not to quantify larval dispersal.

Simulations were run independently per year, with data for 2002 to 2012. The resulting aggregated trajectories allowed the production of connectivity matrices between all pairs of cells. Paired probabilities were inferred by determining the number of steps in the path of a single particle, released from cell i, that achieved cell j, divided by the overall simulated time steps (18 days PLD * 24 steps a day). The annual matrices resulting from the different experiments were averaged to account for interannual variability. This allowed the determination of the maximum and average distance travelled by particles connecting pairs of cells. The mean proportion of retention and effective settlement were also determined and differences between experiment types were tested using a Kruskal-Wallis Rank Sum test followed by a post-hoc pairwise test for multiple comparisons of mean rank sums (Nemenyi-test). Lagrangian dispersal simulations were developed in R [45] using the packages: abind, calibrate, doParallel, igraph, ncdf4, parallel and raster.

## Results

### Genetic diversity

In total, 317 adult and 79 juvenile *L*. *lepadogaster* with a mean standard length of 51.2±8.8 mm and 18.2±4.1 mm, respectively, were successfully genotyped for 12 polymorphic microsatellites. The estimated proportion of population analyzed ranged from 7.0 to 54.9% (Table 1). The number of alleles per locus ranged from 9 (loci LP3, 4, and 15) to 41 (locus LP24). Allelic diversity was very similar between populations as the mean number of alleles across loci varied from 7.8 to 10.3 (Table 2). Allelic diversity of recruits was similar to that of adult populations. Mean allelic richness (A_rich_), standardized to 24 individuals as the minimum common sample size, ranged from 7.5 (LIS_A12, n = 40) to 10.0 (LIS_A11, n = 29). Unbiased expected (H_E_) and observed heterozygosity (H_O_) ranged from 0.72 to 0.81 and 0.56 to 0.74, respectively. All populations had low but significantly positive *F*_*IS*_ values, indicating a departure from Hardy-Weinberg equilibrium (HWE; heterozygote deficit) (Table 1; Table A in S1 File).

**Table 2:** Genetic difference among adult, recruit populations and years.

	**Mar_A12**	**Pen_A12**	**Lis_A12**	**Arr_A12**	**Sin_A12**	**Alm_A12**	**Bar_A12**	**Lis_A11**	**Arr_A11**	**Arr_R12**	**Arr_R11**
**Mar_A12**	--	0.001	-0.003	0.002	0.006*	0.007	0.030***	0.037***	0.048***	0.006*	0.023***
**Pen_A12**	0.024	--	0.000	0.006*	0.009**	0.008*	0.033***	0.038***	0.047***	0.003	0.025***
**Lis_A12**	-0.001	0.015	--	0.004	0.006*	0.003	0.029***	0.039***	0.046***	0.003	0.019***
**Arr_A12**	0.012	0.027	0.02	--	0.004	-0.002	0.023***	0.038***	0.051***	0.005	0.023***
**Sin_A12**	0.043**	0.035**	0.024*	0.024	--	0.009*	0.025***	0.045***	0.053***	0.012**	0.024***
**Alm_A12**	0.043**	0.052**	0.022	0.003	0.034*	--	0.032***	0.035***	0.046***	0.004	0.027***
**Bar_A12**	0.112**	0.109**	0.086**	0.074**	0.080**	0.081**	--	0.055***	0.067***	0.036***	0.041***
**Lis_A11**	0.139**	0.150**	0.152**	0.153**	0.175**	0.146**	0.219**	--	0.022***	0.046***	0.037***
**Arr_A11**	0.138**	0.121**	0.119**	0.123**	0.128**	0.140**	0.189**	0.099**	--	0.051***	0.011**
**Arr_R12**	0.038*	0.020	0.020	0.010	0.038*	0.020	0.106**	0.177**	0.131**	--	0.023***
**Arr_R11**	0.072**	0.070**	0.057**	0.064**	0.064**	0.080**	0.117**	0.138**	0.03	0.055**	--

Pairwise *F*_*ST*_ (above diagonal) and D-values (below diagonal); significance levels are indicated for permutation tests (*p<0.05, **p<0.01, ***p<0.001) and underlined *F*_*ST*_ values are significant also when considering confidence intervals. Population labels are explained in Table 1.

The null allele frequency per locus and population ranged from 0 to 0.27 (Table A in S2 File). Global *F*_*ST*_ for all loci and pairwise *F*_*ST*_ were very similar, either when estimated from raw allele frequencies or from allele frequencies corrected for null alleles (ENA correction of Chapuis and Estoup ([48]; Table B and Table C in S2 File). This suggests that null alleles have very little influence on our analysis; therefore all further tests were performed with uncorrected allele frequencies.

### Population Structure and Connectivity

Global *F*_*ST*_ and Jost’s D value were 0.0227 and 0.0762, respectively. Pairwise *F*_*ST*_ and Jost’s D values showed very similar significant differences between populations (Table 2). Fewer *F*_*ST*_ values were significant, when considering 95% confidence intervals (Table 2). *F*_*ST*_ and D values ranged from -0.003 to 0.067 and -0.001 to 0.189, respectively. D values are less affected by diversity and sample size and were in better concordance with other analyses. According to D-values, in 2012 the most southern population (BAR) was significantly different (p<0.01) from all other populations and SIN was different to all populations north of ARR. In general, there was an increasing differentiation with distance, which coincided with the significant result of the isolation by distance analysis with pairwise *F*_*ST*_ and D values (p = 0.0087 and p = 0.0015, respectively; S4 File in supporting information). Adult individuals from ARR and LIS in 2011, were significantly different (p<0.01) from each other and were also significantly different from both the same populations sampled one year later and from all other populations sampled in 2012. The ARR recruits from 2012 were significantly different (p<0.01) to the most northern population (MAR) and also to SIN and BAR in the south. The 2011 recruit population at ARR was significantly different from all other populations except for the 2011 adults sampled at the same site. D-values were higher for year to year comparisons than site to site, as the D-values of LIS and ARR between 2011 and 2012 were 0.152 and 0.123, respectively and so clearly higher than the D value of MAR and BAR that are separated by 700 km of coastline (0.112, highest D-value among 2012 populations).

**Fig 2:**
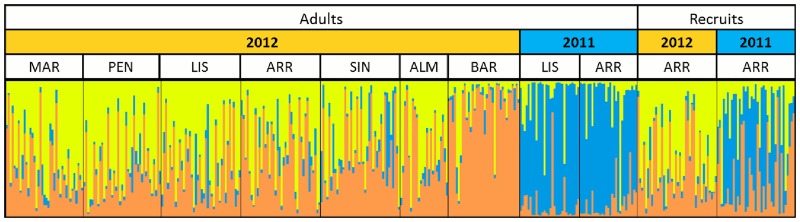
Averaged bar plot of STRUCTURE Bayesian clustering with K = 3. Vertical bars displayed for each individual show the estimated membership proportions in the three clusters colored in yellow, blue and orange. Populations are separated by black vertical lines and described by site abbreviations as in Fig 1, sampling year, and are divided in adult and recruit populations.

The STRUCTURE analysis revealed 3 clusters using L(K) and ΔK methods. All studied populations contained these three genetic populations in different proportions (Fig 2). The composition was very similar among adult populations in 2012, except for the most southern population (BAR). There was a clear difference between populations from 2011 and 2012. The 2012 recruit population matched better with the 2012 adult populations, and the 2011 recruit population matched better with the 2011 adult populations (Fig 2).

**Fig 3:**
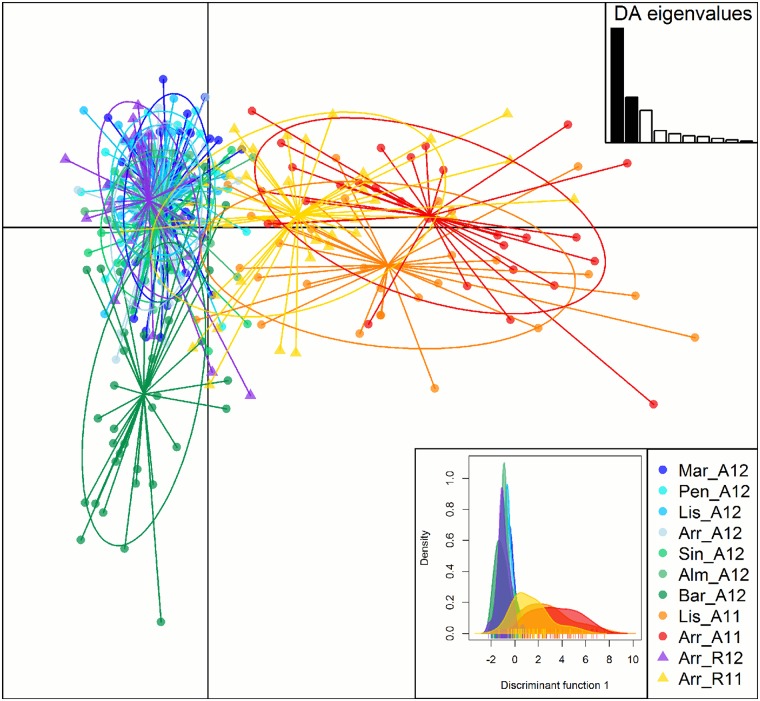
Scatterplot of the DAPC. Individual adult genotypes are represented in dots and recruit genotypes in triangles. Populations are distinguished by colors and 95% inertia ellipses. Barplot of DA eigenvalues (bottom right) displays the proportion of genetic information comprised in each consecutive discriminant function. X and Y axis of the scatterplot describe the first and second discriminant function (explaining 81.8% and 31.6% of genetic variance, respectively). Left from the legend is a density plot of the genetic information from only the first discriminant function.

A similar pattern could be described from the DAPC analysis, whereby 2011 and 2012 populations were differentiated along the first discriminant function that comprised most of the genetic variation (Fig 3). This temporal distinction was even more apparent when densities were plotted only along the first discriminant function (Fig 3, top-right). At the second discriminant function (represented by the y-axis), the most southern location (BAR) stood out from the other 2012 populations, and, to a lesser extent, LIS adults were separated from ARR adults in 2011. The two recruit populations were separated along the first discriminant function. Overall, 61% and 81% of adults were correctly assigned to their geographical population in 2012 and 2011, respectively (Fig B in S3 File), when the recruit populations were excluded. When included, 47% and 62% of adult fish were correctly assigned. Both the STRUCTURE and the DAPC analyses showed that there is more difference between adult populations in 2011 and 2012 for the same sites than among spatially separated populations from the same year.

### Assignment test and parentage analysis

The assignment test detected only very few significant assignments, supporting low genetic structure among these populations. From the 2012 recruits collected at ARR, one was assigned to the population of BAR and one immigrant was not assigned to any of the analyzed populations. In 2011, one recruit was assigned to LIS and another one to ARR.

The parentage analysis identified 6 and 8 parent-offspring pairs in 2011 and 2012, respectively. In 2011 parents originated from ARR (3), SIN (2) and ALM (1) and in 2012 from LIS (5), ARR (1) and ALM (2). This means that over these two years, 5% of recruits came back to their natal population (ARR) and, 13% had a parent from either the next northern or the two next southern populations sampled. Recruits that self-recruited to ARR in 2011 (n = 3) were identified to be the offspring of just one adult fish with a 100% probability. Also, 4 out of the 5 recruits originating from LIS in 2012 shared a parent.

### Dispersal model

**Fig 4:**
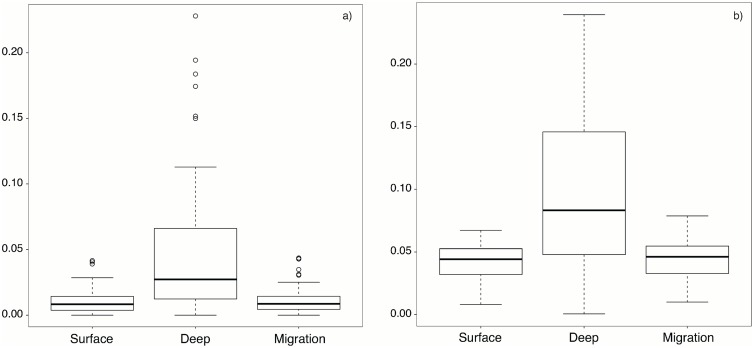
Comparison of particle type. Probability of (a) retention and (b) effective settlement according to particle type (surface, deep and migration).

The numerical simulations using HYCOM velocity fields for an entire 11-year period allowed us to track the fate of 7,920 particles released from 93 distinct coastal cells (736,560 particles in total). Trajectories were obtained for particles that had successfully settlement in any site within 18 days of PLD and these resulted in two types of connectivity matrices: 1) on a yearly basis including 2010, 2011, and 2012 and 2) averaged for an 11-year period. Simulations ran over 11 years revealed that particles spending their entire pelagic phase in a deeper water layer (10 m) had a significantly higher probability of retention compared to particles staying at the surface, or migrating vertically from the surface to the bottom layer at the 10th day of PLD, when the larvae acquired competence for settlement [70] (Fig 4A; Kruskal-Wallis and Nemenyi test, p<0.001). Also, particles that stayed at the bottom layer (10 m) had a significantly higher probability of effective settlement compared to the other two particle conditions (Fig 4B; Kruskal-Wallis and Nemenyi test, p<0.001). This means that by staying near the bottom, more particles are locally retained and are also able to find suitable habitat for settlement during their PLD. Particles that stayed at the surface at the beginning or for the whole pelagic phase had a higher probability of being advected offshore and were thus excluded from the model. Both the probability of retention and effective settlement were not significantly different between surface and migration particles (Kruskal-Wallis and Nemenyi test, p>0.05).

**Fig 5:**
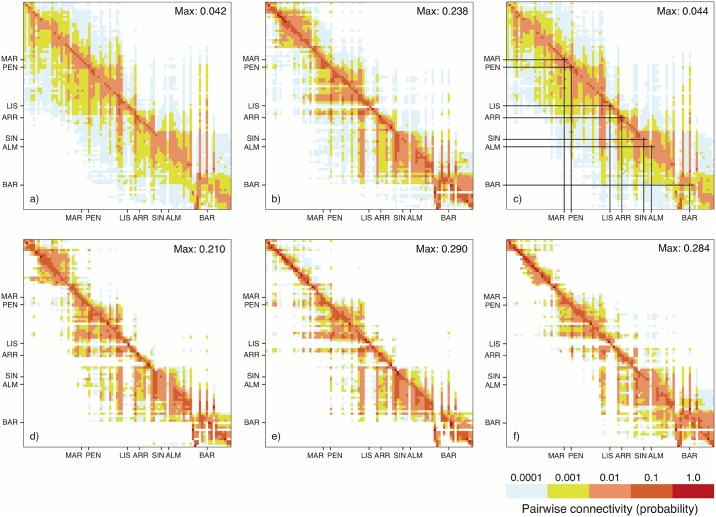
Connectivity matrices for the SW Iberian coast. Displayed in color are averaged probabilities of particle released in site i (y-axis) that settle to site j (x-axis); Simulations ran from 2002 to 2012 for each particle type: surface (a), deep (b) and migration (c) and yearly averaged for the deep ones for 2010 (d), 2011 (e) and 2012 (f). The diagonal represents self-recruitment, cells left of the diagonal show northward dispersal and right of the diagonal southward dispersal. Value on the top right corner indicates the connection made with the highest probability. Axis labels indicate genetic sample sites. First scale color (blue) reaches from a probability of 0 to 0.001 and the last (red) from 0.1 to 1.0.

Particles in deeper waters had an average dispersal capacity of 89.8±70.1 km compared to 105.9±77.0 km and 104.5±75.8 km, for surface particles and vertically migrating particles, respectively. For all three particle types, probability of connectivity declined exponentially with increasing coastline distance, with deep particles having the fastest decline (Fig A in S5 File). The longest distance undertaken by a larva that remained near the bottom was 378.0 km. However, such dispersal events were very rare, as seen in the connectivity matrices (Fig 5A, 5B and 5C and Table A in S5 File).

Connectivity matrices averaged over simulations ran from 2002 to 2012 (Fig 5A, 5B and 5C) also showed higher probabilities of retention (more orange and red cells along diagonal) and shorter dispersal distances (narrower spread around diagonal) for particles staying in deeper waters. In general, these matrices indicated a higher probability of effective dispersal northwards rather than southwards. Furthermore, the 11-year connectivity matrices showed that all sites sampled for genetic analysis were connected by larval dispersal despite low probabilities of connectivity.

Yearly connectivity matrices for 2010, 2011 and 2012 for particles staying in deeper water, a behaviour expected to occur in our study species, showed less spread along the diagonal, indicating reduced long-distance connectivity, when analyzing each year separately (Fig 5D, 5E and 5F). However, interannual differences in dispersal patterns were apparent, with more particles being advected northwards (left from the diagonal) in 2010 and 2011, whereas in 2012 more southward dispersal (right from the diagonal) occurred. Connectivity matrices showed that around the most southern location (BAR) less dispersal occurred northwards, suggesting a connectivity barrier around Cape São Vicente.

When results of the dispersal model were summarized for the whole area of the MPA, the probability of particles settling to that area was 99 and 68 times higher, in 2010 and 2011, respectively, when particles originated from southern sites than for particles released from northern sites (Fig B in S5 File). However, in 2012 this difference was only 10 times higher for southern particles. Particles that were released from the MPA in 2010 and 2011 had 32 and 28 times higher probability, respectively, to settle to northern sites than to southern sites. In all three years the probability of particles being retained in the MPA was 11 to 15 times higher than for particles arriving from southern sites.

## Discussion

This study revealed high genetic connectivity among *Lepadogaster lepadogaster* populations throughout the study area, contradicting our hypothesis of high larval retention in natal areas. However, connectivity decreased with coastline distance and this could be explained by oceanographic effects. The large temporal variability was an unpredicted finding; population differentiation and patterns of dispersal were highly variable between years and this could be explained by interannual variation in current patterns. These results highlight the important role of oceanographic conditions in mediating recruitment patterns despite biological traits which suggested a high level of local recruitment. Larval depth distribution also seems to be an important factor influencing connectivity, as modelling results showed that remaining deeper significantly restricted dispersal distance.

### Spatial scale

In this study, significant heterozygote deficits were found for all populations. A departure from HWE does not affect our analyses, as found in previous studies [71, 72]. The heterozygote deficiency found here can be caused by several factors such as a Wahlund effect, inbreeding or null alleles. Sampling more than one genetic population and treating it as one can result in a Wahlund effect. For our data little spatial sub-structuring was found within populations. However, temporal variation in the annual pool of recruits could result in distinct genetic populations co-occurring and in consequently significant *F*_*IS*_. High inbreeding levels are not expected to occur since *L*. *lepadogaster* can reach high densities and exhibit polygamy. Additionally, the high population genetic diversity and low spatial population structuring found in our study does not support the occurrence of inbreeding leading to a heterozygote deficit.

Another reason for significant *F*_*IS*_ could be null alleles. Estimated null allele frequencies were below 0.2, except for two combinations of locus x population (p<0.27; Table A in S2 File), indicating that null alleles were uncommon to rare [73]. Null alleles did not affect our genetic analyses of population differentiation, since pairwise *F*_*ST*_ for both the original and corrected datasets were similar. The DAPC analysis, that does not require populations to be in HWE [59], showed a similar differentiation as the STRUCTURE analysis. Regarding the parentage analysis, although null alleles could result in an underestimation of the average exclusion probability at a locus, this effect has been shown to be negligible at frequencies below 0.2 [64, 73]. Moreover, since null alleles can lead to a false exclusion of parentage [64, 73], we can expect an underestimation of parent offspring pairs rather than an overestimation in our study.

Using 12 highly polymorphic genetic markers we inferred that populations of *L*. *lepadogaster* were genetically similar along 300 km of coastline and consequently highly connected. Seven out of eight populations from 2012 were placed in one single genetic cluster and genetic diversity was very similar among the 2012 adult populations. Our biophysical model for the SW Iberian coastline supported these results by confirming that all sites sampled for genetics can be connected by dispersal and that it is possible for *L*. *lepadogaster* larvae to disperse long distances over hundreds of kilometers.

Despite the high connectivity estimated, the dispersal model revealed higher probabilities for larval retention and exponentially declining probabilities of dispersal with distance. This is in agreement with the significant isolation-by-distance result obtained. The parentage analysis, a direct method used to estimate connectivity, identified 14 parent-offspring pairs that showed either self-recruitment to ARR or larval dispersal from the nearest sampled populations. From these parent-offspring pairs, 50% of recruits in 2011 and 13% in 2012 self-recruited to ARR.

Both dispersal and retention play an important role in the replenishment of *L*. *lepadogaster* populations, as shown by our results from both indirect and direct measurements of connectivity, which revealed not only high connectivity and possible long-distance dispersal, but also significant levels of self-recruitment and higher probabilities for short-distance dispersal. These results also support the hypothesis that local recruitment is not incompatible with long-distance dispersal and that they may often occur together in marine species (e.g. [74, 75]).

This study further demonstrates that it can be misleading to make assumptions on the degree of population connectivity based solely on a few known early life history traits, such as egg type and PLD. This supports previous studies that showed either higher than expected connectivity (e.g. [10]) or, on the contrary, small scale genetic differentiation where genetic homogeneity was expected (e.g. [76]). Indeed, some genetic studies have found no relation with either egg type (planktonic vs. demersal) and/or PLD for several reef fish species (e.g. [77, 78]).

Larvae of *L*. *lepadogaster* have a low critical swimming speed and it is hypothesized that strong swimming abilities are not needed since they can be retained nearshore on the benthic layer where currents are expected to be weaker due to bottom friction [70]. However, nearshore currents in the study site can be much faster than the registered critical swimming speed (unpublished data). Generally, hydrodynamics in the nearshore area are complex due to the effects of wind, tides, buoyancy, waves and nearshore currents predominantly flowing parallel to shore that can be shifted to nearshore and offshore water exchanges (reviewed in [79]). Thus, specific coastal hydrodynamic conditions could likely allow a significant number of *L*. *lepadogaster* larvae to disperse and eventually reach other populations. This would contribute to a flattening of genetic differences among them, as suggested for a functional dispersal group of organisms with longer dispersal distances, estimated from genetics [80].

### Temporal scale

All analyses in this study revealed strong temporal genetic divergence, with adult gene pools having significantly different compositions between years. This annual difference was even larger than the spatial genetic difference between the two most distant sites (700 km). Moreover, the spatial genetic divergence was also different between years. Although in 2012 all populations (excluding the most southern one: BAR) had a very similar genetic composition, in 2011 the nearby populations of LIS and ARR were more differentiated from each other.

Our findings of temporal variability suggest caution in the interpretation of single dispersal estimates, even when using genetic data which tend to integrate temporal scales. Few genetic studies on reef fish included temporal comparisons and those that did, mostly showed no interannual differences [9, 81, 82], except for Hogan et al. [83, 84]. Our results do not suggest that these differences are caused by either a bottleneck or sweepstake effect (i.e. only a subset of the adult population successfully contributing to subsequent recruitment) since the allele diversity of the recruits was similar to that of the adults [22, 85]. Also, we do not attribute this interannual difference to the effect of small sample sizes as the estimated proportion of the population sampled at LIS was very large (41.2% and 54.9% in 2011 and 2012, respectively).

We propose two possible hypotheses to explain this strong interannual difference in the adult gene pools: i) oceanographic transport; and ii) density dependent recruitment, influenced by storms. The first could be due to differences in coastal currents during the spawning season of *L*. *lepadogaster*, which could lead to larval pools arriving to adult habitats from different source populations in different years [22, 86, 87]. For example, in 2011 parents other than those originating from ARR were identified by parentage analysis to belong to the populations of SIN and ALM, whereas in 2012 most parents were from LIS. These interannual differences match significant differences in the genetic composition of recruits sampled in ARR between 2011 and 2012. This result was consistent in all population genetic analysis and also in the dispersal model, which showed differences in the origin of recruits settling to ARR between those two years.

During late spring and summer months, in addition to the predominant upwelling derived southward currents, inner-shelf northward counter-currents transporting warm water can occur from the south of Portugal to ARR [38]. These could lead to northward transport of larvae and could explain the assignment of recruits sampled in ARR as originating from SIN and ALM and even to the most southern population BAR (assignment test). Connectivity matrices retrieved from our dispersal model showed that successful settlers would mostly arrive from the next southern population, but interannual differences in dispersal patterns were observed when comparing 2010, 2011 and 2012 connectivity matrices individually. In 2010 and 2011 more particles were advected northwards, whereas in 2012 more southward dispersal occurred compared to both previous years. When focusing just on the probability of larvae settling to ARR, the observed year-to-year differences suggest that variable current patterns could cause the variation of genetic composition in recruit populations. Similarly, genetic structure and connectivity among adult populations of a Caribbean reef fish changed over time due to yearly differences in local retention and dispersal patterns [83, 84]. Interannual differences in local retention and self-recruitment were also reported for a coral reef fish in the Red Sea by Nanninga et al. [88].

The second hypothesis to explain the observed temporal differences could be an effect of storms combined with density dependent recruitment success. *L*. *lepadogaster* occurs in discrete patchy habitats [89]. In these conditions, populations can be relatively stable over time as older fish might occupy most of the available space and recruits might have a low probability of successful settlement due to density dependent mechanisms. However, this stability could collapse if a significant number of old fish would die making habitat available for new recruits to settle and influence the population. The LIS adult individuals sampled in 2012 were significantly smaller than in 2011, suggesting that the population in 2012 consisted of younger fish. But, no differences in size were observed for the ARR adult populations. Mortality of a large proportion of the adult population would not be unexpected due to the end of the natural life span or due to winter storm events resulting in strong habitat disturbance. Indeed, at the end of October and beginning of November 2011, extreme storms occurred with wave heights of 4.5 to 5.5 m near LIS and ARR (WANA model [90]; S6 File in supporting information). A storm event causing high mortality of *L*. *lepadogaster* fish could thus be an explanation for the decrease of allelic diversity in the adult populations from 2011 to 2012.

Results of the dispersal model were consistent with the genetic results [20, 91]. However, a limitation of most biophysical models lies in correctly representing nearshore hydrodynamics [92]. Future studies on reef fish ecology directed at analyzing fine-scale current conditions in the reef and looking at alongshore currents nearshore are needed to help clarify the above hypothesis. Furthermore, in a future study a higher number of adult fish should be sampled, since it might enhance the number of parent-offspring pairs revealed by the parentage-analysis [93], as well as the accuracy of the allele frequencies in the populations. Compared to the parentage-analysis, the assignment test detected only very few dispersal trajectories, thus it might lack power to assign recruits correctly when used in a high gene flow scenario despite having highly polymorphic loci (~10; e.g. [74, 93, 94]).

Overall, the genetic population structure supported by the dispersal model indicate significant gene flow due to long-distance larval dispersal that would lead to a homogenization of allele frequencies among sites over an evolutionary time scale. However, the occurrence of temporal genetic divergence in adult and recruit populations suggests that *L*. *lepadogaster* populations might be strongly affected by short-term events such as storms and that populations are highly influenced by the larval pool composition recruiting to the adult habitats. These larval pools would need to be a mixture of fish originating from a variety of source populations to allow for a decrease in the spatial genetic structure within years. But larval pool composition might differ interannually, due to stochastic processes such as highly variable alongshore currents, resulting in adult populations that differ genetically from the previous year.

The *L*. *lepadogaster* population of ARR is not as closed as expected; not only does it depend on the supply of recruits from other populations but it can also provide other sites with larvae, thus functioning simultaneously as a source and sink. A long-term analysis of adults and recruits from all populations over several years would be ideal to follow the temporal dynamics of these source-sink patterns, something that cannot be revealed in studies focused on a single sampling event. The results of this study show that these temporal scales are of great importance in understanding connectivity patterns of coastal reef fish species, thereby providing important information for the design of a network of protected areas.

## Supporting Information

S1 File Summary statistics. Summary statistics for adult and recruit samples of *Lepadogaster lepadogaster* collected along the Portuguese west coast. Shown are expected and observed heterozygosities (H_E_ and H_O_), heterozygote deficiency (FIS) and p-values of FIS for each population and loci.
(PDF)

S2 File FreeNA results—checking for null alleles. **Table A in S2 File.** Estimated null allele frequency for sampled populations (first row) at each locus (first column). **Table B in S2 File.** Comparison of global *F*_*ST*_ per locus derived from raw microsatellite data and after applying the excluding null alleles (ENA) correction method from [48]. **Table C in S2 File**. Comparison of pairwise *F*_*ST*_ derived from raw microsatellite data and after applying the excluding null alleles (ENA) correction method from [48].
(PDF)

S3 File Supporting information. Discriminant analysis of Principal Components (DAPC). **Fig A in S3 File.** Principal Component Analysis of the DAPC; red line indicates number of PCs retained (52) for the DAPC and the genetic information comprised by this number. **Fig B in S3 File**. Correct assignment of adult fish to their geographical population in 2012 and 2011, with recruits being excluded from the DAPC.
(PDF)

S4 File Isolation by distance analysis. Linear regression analysis of genetic distance, estimated from pairwise *F*_*ST*_ (A) and Jost’s D values (B) as a function of shoreline distance.
(PDF)

S5 File Dispersal Model. **Fig A in S5 File.** Coastline distance related to the probability of connectivity averaged over simulations ran from 2002–2012 per particle type surface (a), deep (b) and migration (c); notice different scales of y-axis. **Table A in S5 File**. Paired probabilities of connectivity among genetic sample sites for particles of type deep, migration and surface, averaged over simulations ran from 2002–2012 and yearly averages for 2010, 2011 and 2012. Shaded rows indicate retention probabilities. **Fig B in S5 File**. Connectivity map with probabilities of where particles settle after being released from the MPA (framed) in 2010 (a), 2011 (b) and 2012 (c) and probabilities of the origin of particles that settled into the MPA in 2010 (d), 2011 (e) and 2012 (f).
(PDF)

S6 File Wave and Wind patterns in winter 2011/12. Pattern of wave height for the area of Lisbon (black line) and the MPA (red line) and wind speed of Lisbon during winter 2011/12, retrieved from an oceanographic model [90]; shaded area indicates a period when very strong storms were registered in Portugal.
(PDF)
