# Supplementary material for: High Interannual Variability in Connectivity and Genetic Pool of a Temperate Clingfish Matches Oceanographic Transport Predictions
Source: PLoS One. 2016 Dec 2;11(12):e0165881. doi: 10.1371/journal.pone.0165881 (PMC5135045; doi:10.1371/journal.pone.0165881)
Supplement: S1 File — Summary statistics for adult and recruit samples of Lepadogaster lepadogaster collected along the Portuguese west coast. Shown are expected and observed heterozygosities (HE and HO), heterozygote deficiency (FIS) and p-values of FIS for each population and loci. (PDF) [file pone.0165881.s001.pdf]

S1 Supporting information. Summary statistics.

Summary statistics for adult and recruit samples of Lepadogaster lepadogaster collected along the Portuguese west coast. Shown are expected and observed heterozygosities (HE and HO), heterozygote deficiency (FIS) and p-values of FIS for each population and loci.

	Lp3	Lp4	Lp9	Lp11	Lp13	Lp14	Lp15	Lp17	Lp20	Lp21	Lp23	Lp24
**MAR_A_12**												
N	38	39	39	39	39	39	39	39	39	39	38	39
H_E	0.773	0.686	0.753	0.757	0.688	0.917	0.519	0.817	0.812	0.678	0.641	0.941
H_O	0.684	0.744	0.821	0.769	0.410	0.872	0.564	0.949	0.692	0.615	0.711	0.744
F_{IS}	0.117	-0.085	-0.091	-0.017	0.407	0.050	-0.089	-0.164	0.149	0.093	-0.111	0.212
p-value (F_{IS})	0.057	0.151	0.105	0.364	0.000	0.085	0.136	0.003	0.014	0.107	0.078	0.000
**PEN_A_12**												
N	39	39	39	39	39	39	39	39	39	39	39	39
H_E	0.753	0.719	0.785	0.710	0.652	0.880	0.526	0.800	0.801	0.776	0.541	0.953
H_O	0.744	0.769	0.718	0.769	0.410	0.718	0.487	0.795	0.744	0.641	0.513	0.846
F_{IS}	0.013	-0.070	0.086	-0.085	0.374	0.186	0.075	0.006	0.072	0.175	0.052	0.113
p-value (F_{IS})	0.347	0.181	0.110	0.137	0.000	0.000	0.185	0.377	0.119	0.010	0.239	0.002
**LIS_A_12**												
N	40	40	40	40	40	39	40	40	40	40	40	39
H_E	0.752	0.722	0.765	0.714	0.682	0.873	0.456	0.783	0.785	0.658	0.550	0.933
H_O	0.750	0.600	0.575	0.725	0.375	0.821	0.450	0.725	0.725	0.525	0.500	0.769
F_{IS}	0.003	0.171	0.250	-0.016	0.454	0.061	0.013	0.075	0.078	0.204	0.091	0.177
p-value (F_{IS})	0.401	0.027	0.001	0.361	0.000	0.088	0.350	0.125	0.115	0.006	0.137	0.000
**ARR_A_12**												
N	40	40	38	40	40	39	40	40	39	40	39	39
H_E	0.768	0.709	0.780	0.738	0.661	0.859	0.440	0.837	0.816	0.722	0.685	0.937
H_O	0.750	0.650	0.737	0.675	0.425	0.692	0.450	0.825	0.615	0.575	0.641	0.692
F_{IS}	0.023	0.084	0.056	0.086	0.360	0.196	-0.023	0.015	0.248	0.206	0.065	0.264
p-value (F_{IS})	0.309	0.157	0.183	0.111	0.000	0.000	0.321	0.319	0.000	0.005	0.199	0.000
**SIN_A_12**												
N	40	40	39	40	40	40	39	40	40	40	40	40
H_E	0.772	0.697	0.835	0.769	0.647	0.832	0.367	0.798	0.852	0.730	0.703	0.941
H_O	0.750	0.675	0.897	0.775	0.525	0.825	0.385	0.725	0.825	0.475	0.675	0.850
F_{IS}	0.029	0.031	-0.076	-0.008	0.191	0.009	-0.050	0.092	0.032	0.352	0.041	0.097
p-value (F_{IS})	0.277	0.299	0.087	0.394	0.023	0.323	0.255	0.088	0.226	0.000	0.263	0.005
**ALM_A_12**												
N	24	24	24	24	24	24	24	24	24	24	24	24
H_E	0.796	0.717	0.786	0.606	0.747	0.818	0.473	0.833	0.794	0.812	0.642	0.941
H_O	0.667	0.792	0.833	0.708	0.500	0.833	0.375	0.958	0.708	0.333	0.708	0.833
F_{IS}	0.166	-0.106	-0.062	-0.174	0.335	-0.019	0.211	-0.154	0.110	0.595	-0.106	0.116
p-value (F_{IS})	0.034	0.140	0.204	0.046	0.001	0.292	0.062	0.011	0.092	0.000	0.139	0.007
**BAR_A_12**												
N	35	36	35	36	35	36	36	35	29	33	36	36
H_E	0.740	0.708	0.791	0.701	0.677	0.871	0.471	0.836	0.750	0.728	0.757	0.914
H_O	0.714	0.750	0.829	0.611	0.400	0.722	0.417	0.800	0.793	0.606	0.722	0.833
F_{IS}	0.035	-0.060	-0.049	0.129	0.413	0.173	0.117	0.044	-0.059	0.170	0.046	0.090
p-value (F_{IS})	0.268	0.129	0.218	0.051	0.000	0.001	0.099	0.191	0.165	0.020	0.214	0.024
**LIS_A_11**												
N	30	30	30	29	28	27	30	29	30	30	30	30
H_E	0.725	0.783	0.862	0.807	0.848	0.934	0.666	0.868	0.815	0.840	0.630	0.970
H_O	0.700	0.700	0.833	0.724	0.607	0.815	0.667	0.793	0.867	0.700	0.533	0.900
F_{IS}	0.035	0.107	0.034	0.104	0.288	0.130	-0.001	0.087	-0.064	0.169	0.156	0.073
p-value (F_{IS})	0.266	0.090	0.219	0.082	0.000	0.005	0.418	0.073	0.147	0.009	0.063	0.011
**ARR_A_11**												
N	29	29	29	29	28	23	29	29	29	29	29	29
H_E	0.790	0.707	0.768	0.710	0.785	0.862	0.641	0.837	0.803	0.742	0.575	0.955
H_O	0.793	0.655	0.724	0.655	0.464	0.652	0.690	0.793	0.690	0.655	0.586	0.621
F_{IS}	-0.004	0.075	0.058	0.078	0.413	0.247	-0.077	0.053	0.143	0.119	-0.019	0.354
p-value (F_{IS})	0.413	0.195	0.204	0.162	0.000	0.000	0.168	0.183	0.030	0.058	0.355	0.000
**ARR_R_12**												
N	40	40	39	40	37	40	40	39	39	40	40	40
H_E	0.798	0.722	0.792	0.685	0.672	0.846	0.480	0.819	0.828	0.763	0.369	0.952
H_O	0.850	0.650	0.641	0.600	0.378	0.700	0.425	0.641	0.590	0.500	0.350	0.425
F_{IS}	-0.066	0.101	0.193	0.126	0.441	0.174	0.116	0.219	0.291	0.348	0.052	0.557
p-value (F_{IS})	0.136	0.103	0.005	0.063	0.000	0.001	0.123	0.002	0.000	0.000	0.186	0.000
**ARR_R_11**												
N	39	39	39	39	33	32	38	39	38	38	39	38
H_E	0.773	0.686	0.793	0.722	0.787	0.780	0.651	0.764	0.811	0.528	0.550	0.963
H_O	0.615	0.539	0.718	0.487	0.788	0.719	0.711	0.718	0.605	0.447	0.359	0.737
F_{IS}	0.206	0.218	0.096	0.328	-0.001	0.079	-0.092	0.061	0.256	0.155	0.350	0.237
p-value (F_{IS})	0.006	0.013	0.080	0.000	0.422	0.096	0.144	0.162	0.000	0.038	0.001	0.000
