# Supplementary material for: High Interannual Variability in Connectivity and Genetic Pool of a Temperate Clingfish Matches Oceanographic Transport Predictions
Source: PLoS One. 2016 Dec 2;11(12):e0165881. doi: 10.1371/journal.pone.0165881 (PMC5135045; doi:10.1371/journal.pone.0165881)
Supplement: S2 File — Table A in S2 File. Estimated null allele frequency for sampled populations (first row) at each locus (first column). Table B in S2 File. Comparison of global FST per locus derived from raw microsatellite data and after applying the excluding null alleles (ENA) correction method from [48]. Table C in S2 File. Comparison of pairwise FST derived from raw microsatellite data and after applying the excluding null alleles (ENA) correction method from [48]. (PDF) [file pone.0165881.s002.pdf]

S2 Supporting information. FreeNA results - checking for null alleles.

Table A in S2 Supporting information. Estimated null allele frequency for sampled populations (first row) at each locus (first column).

Locus \ Pop	Mar_A12	Pen_A12	Lis_A12	Arr_A12	Sin_A12	Alm_A12	Bar_A12	Lis_A11	Arr_A11	Arr_R12	Arr_R11
**Lp3**	0.031	0.000	0.001	0.000	0.000	0.048	0.000	0.010	0.000	0.000	0.086
**Lp4**	0.000	0.000	0.051	0.028	0.000	0.000	0.000	0.073	0.006	0.050	0.077
**Lp9**	0.000	0.024	0.093	0.034	0.000	0.007	0.000	0.000	0.057	0.080	0.055
**Lp11**	0.000	0.023	0.000	0.043	0.000	0.000	0.031	0.036	0.039	0.063	0.141
**Lp13**	0.155	0.140	0.176	0.127	0.072	0.122	0.169	0.121	0.162	0.176	0.022
**Lp14**	0.036	0.089	0.035	0.075	0.000	0.000	0.089	0.051	0.091	0.076	0.000
**Lp15**	0.000	0.005	0.000	0.000	0.000	0.063	0.000	0.033	0.000	0.076	0.047
**Lp17**	0.003	0.000	0.006	0.018	0.020	0.000	0.023	0.041	0.008	0.099	0.017
**Lp20**	0.073	0.008	0.025	0.095	0.000	0.029	0.000	0.000	0.075	0.121	0.100
**Lp21**	0.061	0.080	0.073	0.098	0.153	**0.264**	0.058	0.058	0.028	0.158	0.028
**Lp23**	0.000	0.009	0.044	0.023	0.010	0.000	0.000	0.067	0.000	0.014	0.123
**Lp24**	0.092	0.054	0.070	0.122	0.047	0.042	0.037	0.025	0.169	**0.268**	0.111

Legend	
(dark grey)	0.2< x
(medium grey)	0.1 < x < 0.2
(light grey)	0.05 < x< 0.1

S2 Supporting Information. FreeNA results - checking for null alleles

Table B in S2 Supporting information. Comparison of global FST per locus derived from raw microsatellite data and after applying the excluding null alleles (ENA) correction method from [48].

Locus	F_{ST} w/o ENA	F_{ST} w ENA
Lp3	-0.003	-0.002
Lp4	0.001	0.001
Lp9	0.007	0.007
Lp11	0.052	0.05
Lp13	0.057	0.041
Lp14	0.011	0.012
Lp15	0.099	0.099
Lp17	0.005	0.005
Lp20	0.007	0.008
Lp21	0.033	0.034
Lp23	0.026	0.025
Lp24	0.003	0.005
All loci	0.023	0.022

S2 Supporting Information. FreeNA results - checking for null alleles
Table C in S2 Supporting information. Comparison of pairwise FST derived from raw microsatellite data and after applying the excluding null alleles (ENA) correction method from [48].

without ENA

	Mar_A12	Pen_A12	Lis_A12	Arr_A12	Sin_A12	Alm_A12	Bar_A12	Lis_A11	Arr_A11	Arr_R12
**Pen_A12**	0.001									
**Lis_A12**	-0.003	0								
**Arr_A12**	0.002	0.006	0.004							
**Sin_A12**	0.006	0.009	0.006	0.004						
**Alm_A12**	0.007	0.008	0.003	-0.002	0.009					
**Bar_A12**	0.03	0.033	0.029	0.023	0.025	0.032				
**Lis_A11**	0.037	0.037	0.039	0.038	0.045	0.035	0.055			
**Arr_A11**	0.049	0.047	0.046	0.052	0.053	0.047	0.068	0.022		
**Arr_R12**	0.006	0.003	0.003	0.005	0.012	0.004	0.037	0.046	0.052	
**Arr_R11**	0.023	0.024	0.018	0.023	0.024	0.027	0.041	0.037	0.011	0.022

with ENA

	Mar_A12	Pen_A12	Lis_A12	Arr_A12	Sin_A12	Alm_A12	Bar_A12	Lis_A11	Arr_A11	Arr_R12
**Pen_A12**	0.002									
**Lis_A12**	-0.003	-0.001								
**Arr_A12**	0.002	0.006	0.004							
**Sin_A12**	0.007	0.009	0.006	0.003						
**Alm_A12**	0.009	0.008	0.005	0	0.009					
**Bar_A12**	0.03	0.033	0.029	0.022	0.025	0.033				
**Lis_A11**	0.032	0.032	0.032	0.032	0.039	0.03	0.049			
**Arr_A11**	0.043	0.042	0.04	0.048	0.05	0.042	0.063	0.021		
**Arr_R12**	0.008	0.005	0.005	0.008	0.013	0.006	0.039	0.039	0.047	
**Arr_R11**	0.022	0.024	0.018	0.022	0.024	0.028	0.039	0.034	0.013	0.024
