# Supplementary material for: High Interannual Variability in Connectivity and Genetic Pool of a Temperate Clingfish Matches Oceanographic Transport Predictions
Source: PLoS One. 2016 Dec 2;11(12):e0165881. doi: 10.1371/journal.pone.0165881 (PMC5135045; doi:10.1371/journal.pone.0165881)
Supplement: S3 File — Fig A in S3 File. Principal Component Analysis of the DAPC; red line indicates number of PCs retained (52) for the DAPC and the genetic information comprised by this number. Fig B in S3 File. Correct assignment of adult fish to their geographical population in 2012 and 2011, with recruits being excluded from the DAPC. (PDF) [file pone.0165881.s003.pdf]

S3 Supporting information. Discriminat analysis of Principal Components (DAPC)

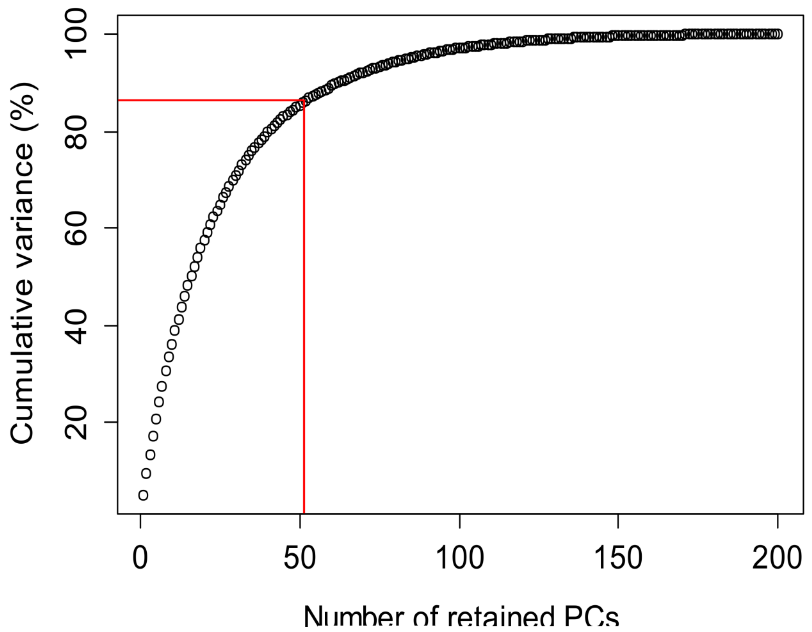

Fig A in S3 Supporting information. Principal Component Analysis of the DAPC; red line indicates number of PCs retained (52) for the DAPC and the genetic information comprised by this number.

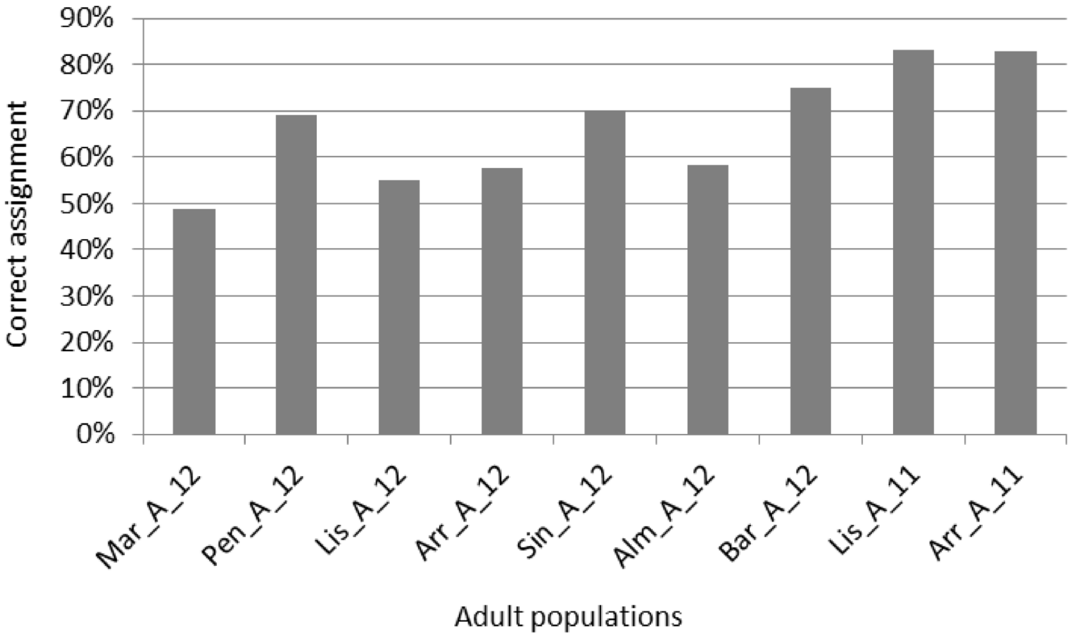

Fig B in S3 Supporting information. Correct assignment of adult fish to their geographical population in 2012 and 2011, with recruits being excluded from the DAPC.
