# Supplementary material for: High Interannual Variability in Connectivity and Genetic Pool of a Temperate Clingfish Matches Oceanographic Transport Predictions
Source: PLoS One. 2016 Dec 2;11(12):e0165881. doi: 10.1371/journal.pone.0165881 (PMC5135045; doi:10.1371/journal.pone.0165881)
Supplement: S4 File — Linear regression analysis of genetic distance, estimated from pairwise FST (A) and Jost’s D values (B) as a function of shoreline distance. (PDF) [file pone.0165881.s004.pdf]

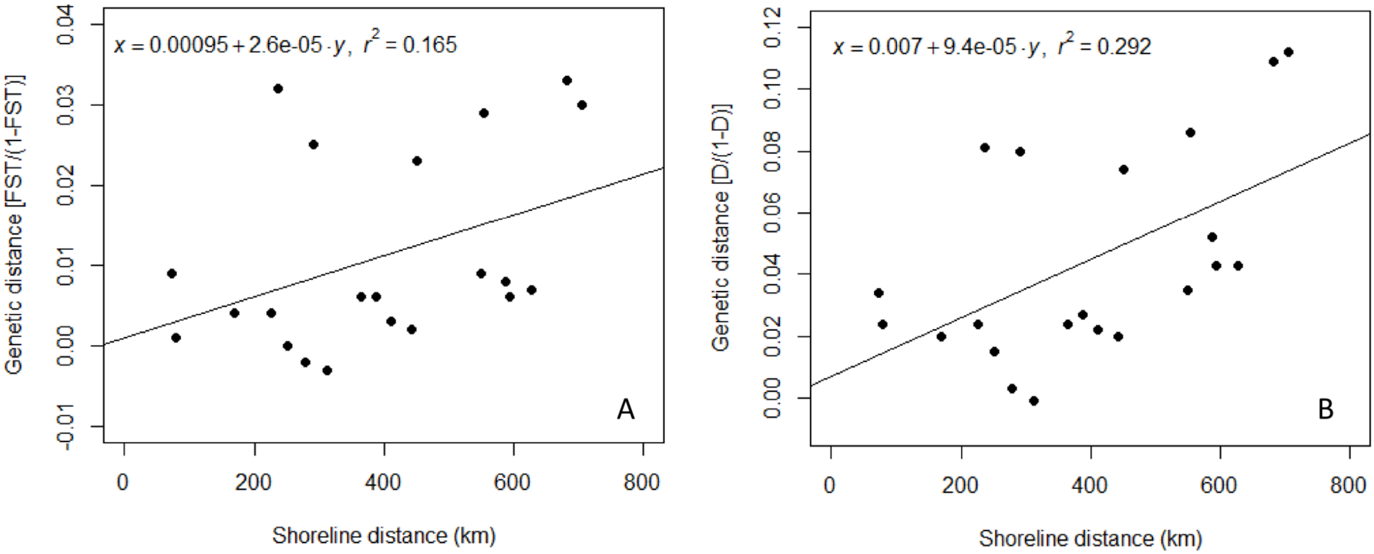

S4 Figure. Isolation by distance analysis. Linear regression analysis of genetic distance, estimated from pairwise FST (A) and Jost's D values (B) as a function of shoreline distance.
