# Supplementary material for: High Interannual Variability in Connectivity and Genetic Pool of a Temperate Clingfish Matches Oceanographic Transport Predictions
Source: PLoS One. 2016 Dec 2;11(12):e0165881. doi: 10.1371/journal.pone.0165881 (PMC5135045; doi:10.1371/journal.pone.0165881)
Supplement: S5 File — Fig A in S5 File. Coastline distance related to the probability of connectivity averaged over simulations ran from 2002–2012 per particle type surface (a), deep (b) and migration (c); notice different scales of y-axis. Table A in S5 File. Paired probabilities of connectivity among genetic sample sites for particles of type deep, migration and surface, averaged over simulations ran from 2002–2012 and yearly averages for 2010, 2011 and 2012. Shaded rows indicate retention probabilities. Fig B in S5 File. Connectivity map with probabilities of where particles settle after being released from the MPA (framed) in 2010 (a), 2011 (b) and 2012 (c) and probabilities of the origin of particles that settled into the MPA in 2010 (d), 2011 (e) and 2012 (f). (PDF) [file pone.0165881.s005.pdf]

S5 Supporting information. Dispersal Model.

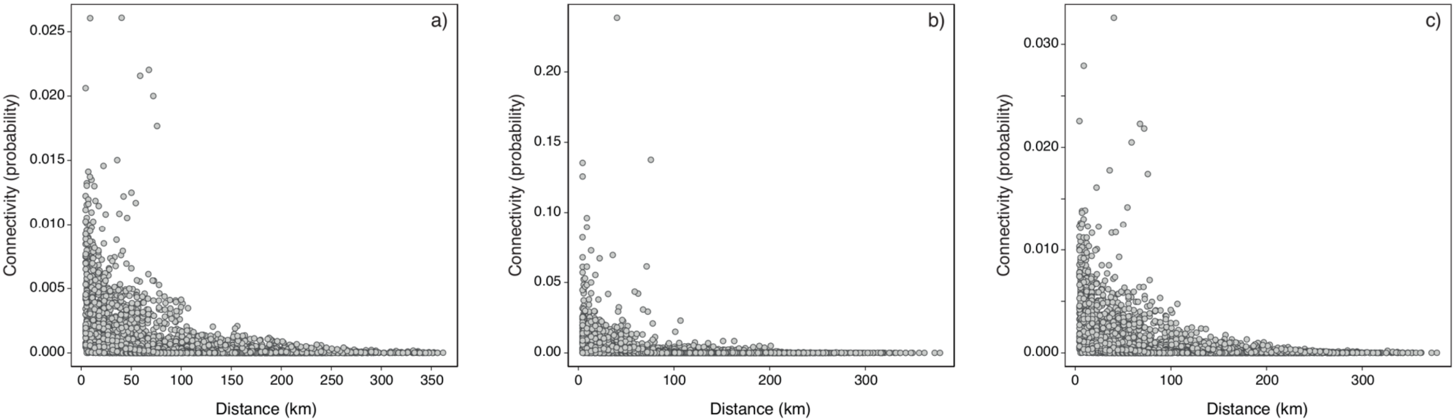

Fig A in S5 Supporting information. Coastline distance related to the probability of connectivity averaged over simulations ran from 2002 – 2012 per particle type surface (a), deep (b) and migration (c); notice different scales of y-axis.

S5 Supporting information. Dispersal Model.

Table A in S5 Supporting information. Paired probabilities of connectivity among genetic sample sites for particles of type deep, migration and surface, averaged over simulations ran from 2002 - 2012 and yearly averages for 2010, 2011 and 2012. Shaded rows indicate retention probabilities.

		Deep				Migration				Surface			
**FROM**	**TO**	**AVG**	**2010**	**2011**	**2012**	**AVG**	**2010**	**2011**	**2012**	**AVG**	**2010**	**2011**	**2012**
MAR	MAR	0.0415	0.0380	0.0815	0.0505	0.0125	0.0142	0.0116	0.0194	0.0127	0.0122	0.0098	0.0176
PEN	MAR	0.0129	0.0048	0.0207	0.0110	0.0028	0.0018	0.0021	0.0008	0.0018	0.0020	0.0024	0.0008
LIS	MAR	0.0003	0.0004	0.0000	0.0000	0.0005	0.0000	0.0009	0.0000	0.0003	0.0000	0.0001	0.0000
ARR	MAR	0.0011	0.0003	0.0010	0.0000	0.0001	0.0000	0.0001	0.0000	0.0001	0.0000	0.0001	0.0000
SIN	MAR	0.0000	0.0000	0.0000	0.0000	0.0000	0.0000	0.0000	0.0000	0.0000	0.0000	0.0000	0.0000
ALM	MAR	0.0000	0.0000	0.0000	0.0000	0.0000	0.0000	0.0000	0.0000	0.0000	0.0000	0.0000	0.0000
BAR	MAR	0.0000	0.0000	0.0000	0.0000	0.0000	0.0000	0.0000	0.0000	0.0000	0.0000	0.0000	0.0000
MAR	PEN	0.0006	0.0004	0.0001	0.0007	0.0022	0.0017	0.0015	0.0015	0.0026	0.0020	0.0010	0.0021
PEN	PEN	0.0144	0.0140	0.0167	0.0139	0.0133	0.0118	0.0097	0.0159	0.0145	0.0120	0.0095	0.0159
LIS	PEN	0.0003	0.0004	0.0000	0.0000	0.0003	0.0008	0.0006	0.0002	0.0003	0.0006	0.0001	0.0000
ARR	PEN	0.0017	0.0030	0.0012	0.0005	0.0004	0.0009	0.0003	0.0000	0.0003	0.0006	0.0003	0.0000
SIN	PEN	0.0000	0.0000	0.0000	0.0000	0.0000	0.0002	0.0000	0.0000	0.0000	0.0003	0.0000	0.0000
ALM	PEN	0.0001	0.0013	0.0000	0.0000	0.0001	0.0006	0.0000	0.0000	0.0000	0.0001	0.0000	0.0000
BAR	PEN	0.0000	0.0000	0.0000	0.0000	0.0000	0.0000	0.0000	0.0000	0.0000	0.0000	0.0000	0.0000
MAR	LIS	0.0000	0.0000	0.0000	0.0000	0.0000	0.0000	0.0000	0.0000	0.0000	0.0000	0.0000	0.0000
PEN	LIS	0.0000	0.0000	0.0000	0.0000	0.0000	0.0000	0.0000	0.0000	0.0000	0.0000	0.0000	0.0000
LIS	LIS	0.0804	0.0670	0.1366	0.0919	0.0108	0.0085	0.0066	0.0070	0.0105	0.0076	0.0075	0.0075
ARR	LIS	0.0033	0.0018	0.0087	0.0019	0.0010	0.0006	0.0003	0.0000	0.0010	0.0006	0.0002	0.0001
SIN	LIS	0.0000	0.0000	0.0000	0.0000	0.0001	0.0004	0.0000	0.0000	0.0000	0.0000	0.0002	0.0000
ALM	LIS	0.0001	0.0000	0.0000	0.0000	0.0001	0.0003	0.0001	0.0000	0.0000	0.0000	0.0000	0.0000
BAR	LIS	0.0001	0.0001	0.0000	0.0000	0.0001	0.0000	0.0000	0.0000	0.0000	0.0000	0.0000	0.0000
MAR	ARR	0.0000	0.0000	0.0000	0.0000	0.0000	0.0000	0.0000	0.0000	0.0000	0.0000	0.0000	0.0000
PEN	ARR	0.0000	0.0000	0.0000	0.0000	0.0000	0.0000	0.0000	0.0001	0.0001	0.0000	0.0000	0.0003
LIS	ARR	0.0003	0.0001	0.0008	0.0000	0.0016	0.0012	0.0014	0.0015	0.0021	0.0012	0.0009	0.0048
ARR	ARR	0.0213	0.0157	0.0205	0.0297	0.0208	0.0158	0.0176	0.0211	0.0209	0.0131	0.0150	0.0189
SIN	ARR	0.0037	0.0051	0.0024	0.0008	0.0050	0.0056	0.0061	0.0062	0.0034	0.0012	0.0031	0.0045
ALM	ARR	0.0113	0.0040	0.0078	0.0135	0.0050	0.0056	0.0056	0.0026	0.0033	0.0026	0.0020	0.0022
BAR	ARR	0.0069	0.0103	0.0080	0.0009	0.0006	0.0010	0.0003	0.0000	0.0004	0.0001	0.0006	0.0000
MAR	SIN	0.0000	0.0000	0.0000	0.0000	0.0000	0.0000	0.0000	0.0000	0.0000	0.0000	0.0000	0.0000
PEN	SIN	0.0000	0.0000	0.0000	0.0000	0.0000	0.0000	0.0000	0.0000	0.0000	0.0000	0.0000	0.0000
LIS	SIN	0.0000	0.0000	0.0000	0.0000	0.0000	0.0000	0.0000	0.0000	0.0000	0.0000	0.0000	0.0000
ARR	SIN	0.0000	0.0000	0.0000	0.0000	0.0000	0.0000	0.0000	0.0000	0.0000	0.0000	0.0000	0.0000
SIN	SIN	0.0623	0.0460	0.1308	0.0315	0.0182	0.0133	0.0225	0.0182	0.0162	0.0126	0.0230	0.0170
ALM	SIN	0.0297	0.0200	0.0753	0.0081	0.0072	0.0054	0.0040	0.0061	0.0061	0.0058	0.0040	0.0049
BAR	SIN	0.0015	0.0007	0.0005	0.0059	0.0002	0.0001	0.0000	0.0000	0.0001	0.0003	0.0000	0.0000
MAR	ALM	0.0000	0.0000	0.0000	0.0000	0.0000	0.0000	0.0000	0.0000	0.0000	0.0000	0.0000	0.0000
PEN	ALM	0.0000	0.0000	0.0000	0.0000	0.0000	0.0000	0.0000	0.0000	0.0000	0.0000	0.0000	0.0000
LIS	ALM	0.0000	0.0000	0.0000	0.0000	0.0000	0.0000	0.0000	0.0000	0.0000	0.0000	0.0000	0.0000
ARR	ALM	0.0000	0.0000	0.0000	0.0000	0.0000	0.0000	0.0000	0.0001	0.0000	0.0000	0.0000	0.0001
SIN	ALM	0.0018	0.0011	0.0019	0.0021	0.0026	0.0040	0.0011	0.0029	0.0023	0.0033	0.0012	0.0028
ALM	ALM	0.0113	0.0090	0.0153	0.0124	0.0061	0.0075	0.0060	0.0054	0.0058	0.0065	0.0074	0.0060
BAR	ALM	0.0023	0.0014	0.0041	0.0023	0.0003	0.0001	0.0007	0.0001	0.0001	0.0002	0.0001	0.0000
MAR	BAR	0.0000	0.0000	0.0000	0.0000	0.0000	0.0000	0.0000	0.0000	0.0000	0.0000	0.0000	0.0000
PEN	BAR	0.0000	0.0000	0.0000	0.0000	0.0000	0.0000	0.0000	0.0000	0.0000	0.0000	0.0000	0.0000
LIS	BAR	0.0000	0.0000	0.0000	0.0000	0.0000	0.0000	0.0000	0.0000	0.0001	0.0000	0.0000	0.0000
ARR	BAR	0.0000	0.0000	0.0000	0.0000	0.0000	0.0000	0.0000	0.0000	0.0005	0.0001	0.0000	0.0008
SIN	BAR	0.0000	0.0000	0.0000	0.0000	0.0002	0.0000	0.0000	0.0002	0.0028	0.0010	0.0003	0.0078
ALM	BAR	0.0000	0.0000	0.0000	0.0000	0.0007	0.0002	0.0001	0.0008	0.0034	0.0023	0.0009	0.0071
BAR	BAR	0.0119	0.0175	0.0123	0.0147	0.0044	0.0021	0.0061	0.0069	0.0030	0.0027	0.0049	0.0009

S5 Supporting information. Dispersal Model.

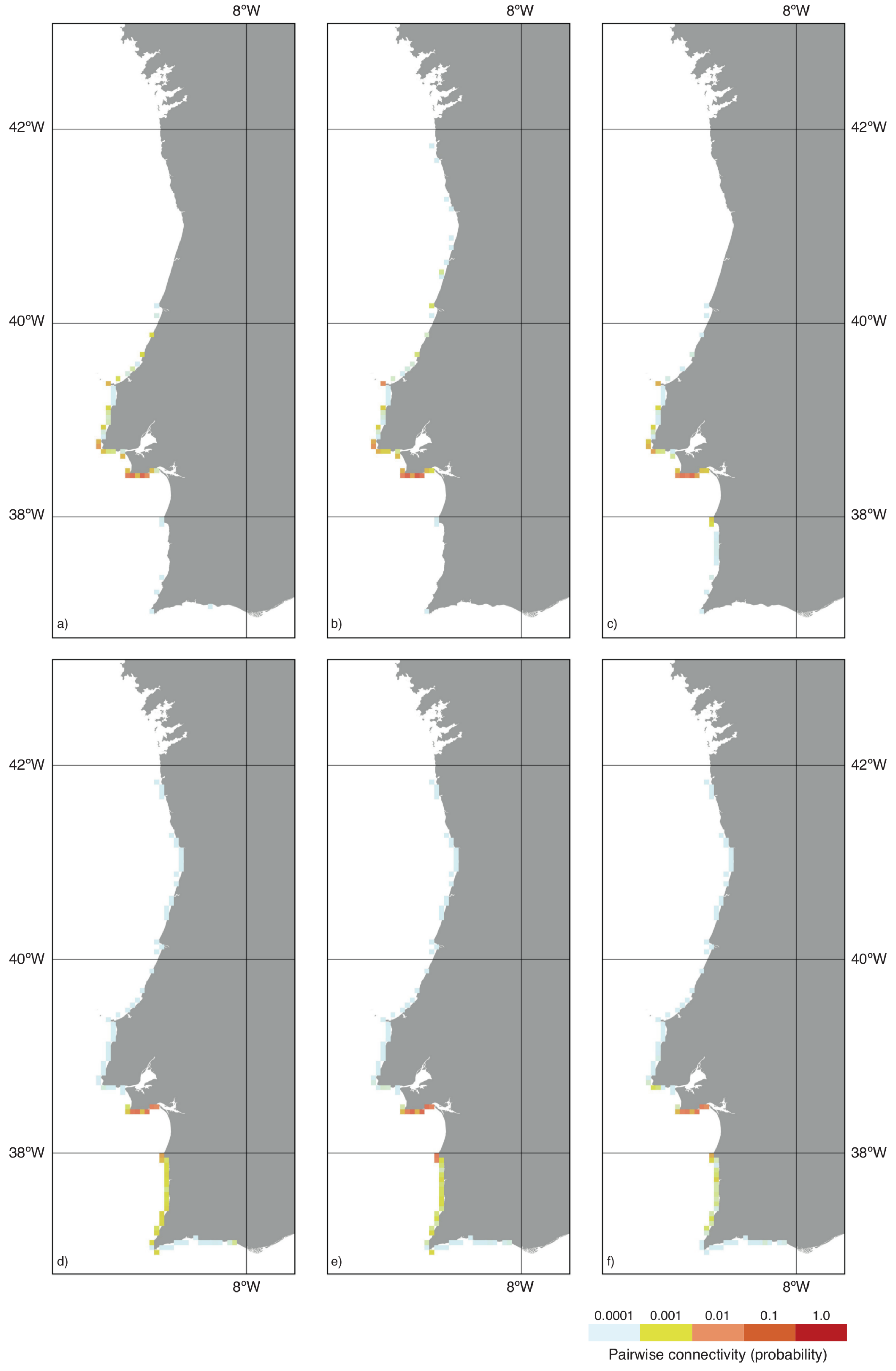

Fig B in S5 Supporting information. Connectivity map with probabilities of where particles settle after being released from the MPA (framed) in 2010 (a), 2011 (b) and 2012 (c) and probabilities of the origin of particles that settled into the MPA in 2010 (d), 2011 (e) and 2012 (f).
