# Supplementary material for: High Interannual Variability in Connectivity and Genetic Pool of a Temperate Clingfish Matches Oceanographic Transport Predictions
Source: PLoS One. 2016 Dec 2;11(12):e0165881. doi: 10.1371/journal.pone.0165881 (PMC5135045; doi:10.1371/journal.pone.0165881)
Supplement: S6 File — Pattern of wave height for the area of Lisbon (black line) and the MPA (red line) and wind speed of Lisbon during winter 2011/12, retrieved from an oceanographic model [90]; shaded area indicates a period when very strong storms were registered in Portugal. (PDF) [file pone.0165881.s006.pdf]

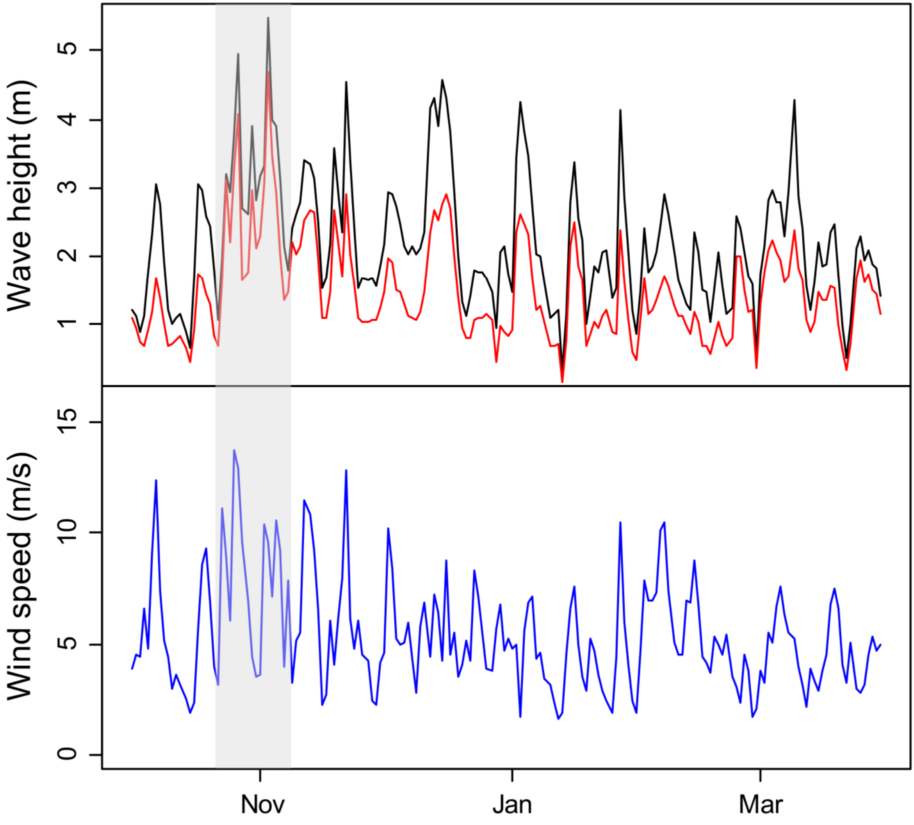

S6 Figure. Wave and Wind patterns in winter 2011/12.
Pattern of wave height for the area of Lisbon (black line) and the MPA (red line) and wind speed of Lisbon during winter 2011/12, retrieved from an oceanographic model [90]; shaded area indicates a period when very strong storms were registered in Portugal.
